# Crosstalk Between Intestinal Microbiota and Host Defense Peptides in Fish

**DOI:** 10.3390/biology14091243

**Published:** 2025-09-11

**Authors:** Xiao-Zheng Yu, Yang Yu, Zi-Yan Liu

**Affiliations:** 1 Key Laboratory of Pollution Exposure and Health Intervention of Zhejiang Province, College of Biological and Environmental Engineering, Zhejiang Shuren University, Hangzhou 310015, China; 601824@zjsru.edu.cn; 2 State Key Laboratory of Biocontrol, Guangdong Province Key Laboratory for Aquatic Economic Animals, Guangdong Provincial Engineering Technology Research Center for Healthy Breeding of Important Economic Fish, School of Life Sciences, Sun Yat-Sen University, Guangzhou 510275, China; yuyang65@mail2.sysu.edu.cn

**Keywords:** host defense peptides, microbiota, crosstalk, sustainable aquaculture, immunology

## Abstract

The intricate crosstalk between intestinal microbiota and host defense peptides (HDPs) in aquaculture has emerged as a cornerstone for advancing sustainable disease management and reducing reliance on antibiotics. This review synthesizes current insights into the bidirectional interactions shaping aquatic animal health, where HDPs, multifunctional immune molecules, directly neutralize pathogens while selectively modulating intestinal microbial communities to favor beneficial taxa (including *Lactobacillus*, *Bacillus*, *Cetobacterium, Lactococcus*, and so on) and suppress harmful species. Conversely, intestinal microbiota regulate HDP expression through microbial-derived signals, such as lipopolysaccharides and metabolites, which activate host immune pathways like Toll-like receptors (TLRs) to amplify innate defenses. This dynamic interplay underpins critical physiological functions, including nutrient absorption, intestinal barrier integrity, and systemic immune homeostasis, offering a dual mechanism to enhance disease resistance and growth performance. Practical applications, such as HDP-enriched feeds and probiotic–HDP synergies, have demonstrated efficacy in reducing mortality and improving productivity across species like shrimp, salmon, and carp. However, challenges such as HDP instability, species-specific variability in peptide efficacy, and the complexity of microbiota–HDP networks hinder broad implementation. Future research must prioritize innovative strategies, including engineered microbial systems for scalable HDP production, multi-omics approaches to unravel interaction mechanisms, and eco-friendly combinatorial therapies integrating HDPs, probiotics, and plant-derived compounds. By bridging immunology, microbiology, and aquaculture science, this field can transition toward antibiotic-free practices, ensuring ecological sustainability and global food security in the face of rising aquatic disease threats and environmental pressures.

## Simple Summary

Fish rely on a healthy intestine to grow well and fight off diseases. Inside their intestines, there is a constant interaction between helpful microbes and special molecules made by the animal called host defense peptides (HDPs). These peptides act like natural antibiotics: they kill harmful bacteria and help promote good ones, such as *Lactobacillus* and *Bacillus*. In return, the intestinal microbiota can influence how many of these peptides are produced by sending signals to the immune system. This two-way relationship helps fish digest food better, maintain strong intestinal barriers, and stay healthy without needing antibiotics. Scientists are now exploring ways to use HDPs in fish feeds or combine them with probiotics to prevent disease more naturally. Although some challenges remain, like keeping HDPs stable and understanding how they work in different species, this approach offers a promising path toward safer, more sustainable aquaculture.

## 1. Introduction

During the last decade, there has been a dramatic growing trend in the aquaculture industry, which played an increasingly vital role in maintaining food security and human nutrition. In 2022, global fisheries and aquaculture production is at a record high, reaching 223.2 million tonnes, which is composed of 185.4 million tonnes of aquatic animals and 37.8 million tonnes of algae [1]. However, the demand for boosted production causes higher stocking density in aquaculture, which stresses the fish, and ultimately raises their risk of disease while suppressing growth and immunity [2,3,4]. The intestinal microbiota is dynamic microbial communities, which are increasingly being considered as crucial modulatory factors for welfare and disease in terrestrials and fish [5,6,7,8]. Once intestinal microbial homeostasis is broken by environmental stress and/or pathogens invasion, it will easily suffer from a series of serious metabolic disorders and diseases such as leaky intestinal syndrome, inflammation, and abnormal fat deposition [6,9,10], illustrating that intestinal dysbiosis can give rise to suppressing nutrient absorption and damaging health of host. Therefore, maintenance of intestinal microbial homeostasis can be instrumental in improving aquatic animal welfare and developing sustainable aquaculture.

Host defense peptides (HDPs), also called antimicrobial peptides (AMPs), are evolutionary conserved components of the innate immune system that were expressed by all taxa. HDPs have been identified as primary modulators of interplay between commensal microbiota and host tissues [11,12]. Interestingly, the antimicrobial activities of HDPs are not necessarily the most primary mechanism in the modulation of intestinal injury. Instead, HDPs may promote certain protective microbial species and modulate innate immune responses, which will be conducive to maintaining intestinal microbial homeostasis [12,13,14].

The gastrointestinal tract relies on a dynamic interplay between Paneth cells and enterocytes, which act as primary sources of HDPs critical for maintaining microbial balance. Paneth cells, strategically positioned in intestinal crypts near stem cells, secrete specialized HDPs like defensins and regenerating family member 3 gamma (RegIIIγ) to directly combat pathogens while shaping the microbiota [13]. Concurrently, goblet cells produce mucin, forming a physical barrier that separates epithelial cells from microbes and prevents pathogen adhesion [15,16]. The integrity of this epithelial barrier is essential for homeostasis [17]; disruptions, such as reduced HDPs production (including β-defensin, hepcidin-1, natural killer (NK)-lysin, and piscidin-5) in intestinal of spotted seabass (*Lateolabrax maculatus*) fed soybean meal diet [18], can lead to dysbiosis and increased susceptibility to infections by pathogens like *Yersinia pseudotuberculosis* or *Pseudomonas aeruginosa* [19,20]. HDPs not only directly inhibit microbial growth but also regulate epithelial repair and stem cell activity, highlighting their dual role in defense and tissue maintenance [21,22]. Additionally, bacterially derived peptides from commensal microbes further contribute to pathogen restriction, underscoring the complex multitiered defense system that safeguards gastrointestinal health [23,24,25].

Understanding the crosstalk between intestinal microbiota and HDPs is critical for sustainable aquaculture, addressing challenges like disease outbreaks, antibiotic overuse, and environmental stress. This bidirectional interaction plays a key role in maintaining intestinal health: microbiota regulate HDPs expression via metabolites and signaling pathways, while HDPs shape microbial composition and suppress pathogens. By elucidating these mechanisms, researchers can develop eco-friendly strategies, such as probiotics and functional feeds, to enhance host immunity and reduce antibiotics reliance. For example, probiotics like *Bacillus siamensis* boost four HDPs production (including β-defensin, hepcidin-1, NK-lysin, and piscidin-5) in spotted seabass (*L. maculatus*), positively shaping intestinal microbiota by increased the abundance of common probiotics (Fusobacteria, Firmicutes, Bacteroidota, and *Cetobacterium*) and decreased opportunistic pathogens including Proteobacteria and *Plesiomonasare* [26,27]. Such innovations align with global efforts to transition toward sustainable aquaculture, reduce the environmental footprint and safeguard consumer health. Additionally, insights into this crosstalk may lead to precision nutrition and novel therapies, fostering resilient aquatic populations and protecting both human health and ecosystems.

While recent reviews have thoroughly cataloged the taxonomic composition and functional potential of the fish intestinal microbiota [28,29,30] and others have comprehensively summarized the structures, mechanisms, and biotechnological promise of fish-derived HDPs [31,32,33], relatively few works explicitly integrate molecular evidence for bidirectional interactions between HDPs and intestinal microbial communities nor translate those mechanisms into practical aquaculture interventions. The present review addresses this gap by (1) synthesizing mechanistic studies that show how HDPs modulate microbiota composition and function (membrane perturbation, quorum-sensing interference, and metabolite-mediated effects) and conversely how microbiota-derived signals regulate HDP expression and activity, and (2) mapping those mechanisms onto actionable, species-relevant strategies for aquaculture, including engineered probiotic platforms, scalable HDP production/delivery systems, and targeted multi-omics workflows to validate efficacy. By focusing on the mechanism-to-application pathway rather than cataloging either domain in isolation, we provide a compact conceptual framework intended to accelerate translational work that bridges innate immunity/HDPs and microbiota in fish. Furthermore, we propose the hypothesis that HDPs act as immune modulators that stabilize beneficial microbiota under farming-related stressors, thereby enhancing both disease resistance and production efficiency

## 2. Overview of HDPs in Fish

Fish inhabit diverse aquatic environments such as rivers, freshwater lakes, and oceans [34]. As one of the oldest vertebrate groups, they share fundamental immune features with higher vertebrates (e.g., birds, reptiles, and mammals), comprising both innate and adaptive immunity [35]. However, unlike mammals, fish are ectothermic, and their survival in dynamic aquatic settings heavily depends on rapid, broad-spectrum innate immune responses rather than slower, pathogen-specific adaptive immunity [36,37]. This reliance is particularly critical given the constant exposure to waterborne pathogens, including bacteria, viruses, and fungi, which thrive in aquatic environments and challenge fish health [38,39,40]. Among the frontline defenders in this immune arsenal are HDPs, small bioactive molecules that act as essential mediators of innate immunity. When fish encounter physical injury or microbial invasion, HDPs are swiftly produced by epithelial cells, phagocytes, and mucosal tissues to neutralize pathogens through membrane disruption, enzyme inhibition, or immune signaling modulation [32,41].

Over decades of research, several major HDP families have been identified in fish, each with distinct structural and functional roles [33]. For instance, hepcidins regulate iron metabolism while exhibiting potent antibacterial and antiviral activity [42,43]; β-defensins target Gram-negative bacteria through membrane permeabilization [44,45]; piscidins, unique to teleost fish, display broad activity against both Gram-positive and Gram-negative pathogens [46]; cathelicidins contribute to wound healing and immune cell recruitment [47]; and NK-lysins, derived from cytotoxic T lymphocytes and natural killer (NK) cells [48], combat parasites and intracellular pathogens [49,50]. These peptides often act synergistically, forming a robust chemical barrier that complements physical defenses like scales and mucus. For example, Larimicin_78-102_, a novel HDP from large yellow croaker (*Larimichthys crocea*), could increase *piscidin*, *hepcidin*, and *lysozyme* expression and relieve inflammation, eventually enhancing the survival rate of large yellow croaker after *Vibrio fluvialis* infection [51]. Their rapid induction and versatility make HDPs indispensable for fish survival, especially in resource-limited or pathogen-rich environments. Furthermore, the evolutionary conservation of these peptides across vertebrates underscores their fundamental role in bridging ancient and modern immune strategies, offering insights for applications in aquaculture disease management and sustainable farming practices.

### 2.1. Definition and Classification

HDPs are small, evolutionarily conserved molecules that serve as vital components of innate immunity in fish. Typically composed of 12–50 amino acids, these peptides are characterized by their cationic (positively charged) and amphipathic structures, allowing them to interact with and disrupt the negatively charged membranes of pathogens. Structurally, HDPs adopt diverse conformations (including α-helices, β-sheets, or extended loops) tailored to their ecological niches and pathogenic pressures. For instance, marine fish HDPs like pleurocidin exhibit enhanced salt tolerance to function effectively in saline environments [52,53,54]. This classification not only reflects evolutionary adaptations but also underscores the functional versatility of HDPs in maintaining host–microbe balance. Beyond direct antimicrobial action, many HDPs modulate immune signaling pathways, enhance barrier integrity, and even influence the composition of beneficial intestinal microbiota. In fish (Table 1), HDPs are primarily classified into families such as hepcidins, β-defensins, piscidins, cathelicidins, and NK-lysins based on their structural features (e.g., amino acid composition, secondary structure, and disulfide bond patterns) and biological functions (e.g., antimicrobial mechanisms, iron regulation, or immunomodulatory roles) [33,40].

Hepcidins, also known as iron-regulatory peptides, are cysteine-rich polypeptides synthesized primarily in the liver, renowned for their broad-spectrum antimicrobial properties [55]. Structurally, hepcidins consist of three domains including an N-terminal signal peptide, a central propeptide region, and a C-terminal mature peptide stabilized by disulfide bonds [56,57]. The propeptide region typically harbors a conserved R-X-K/R-R motif at its C-terminus, which serves as a cleavage site for prohormone convertases during post-translational processing [58]. Phylogenetic analyses of fish hepcidins have classified them into HAMP1 (hepcidin antimicrobial peptide 1 type) and HAMP2 [59]. HAMP1widely distributed across fish species and sharing high homology with mammalian hepcidins, and HAMP2 is exclusive to acanthopterygians (spiny-finned fishes) [59,60]. The mature peptide of HAMP1 features an iron-regulatory sequence (Q-S-H/N-L/I-S) at its N-terminus, which plays a critical role in iron transport and regulation, whereas this motif is absent in HAMP2, suggesting functional divergence [61,62]. Beyond their role in iron homeostasis, fish hepcidins exhibit potent activity against Gram-positive and Gram-negative bacteria, fungi, and viruses, as demonstrated in diverse studies [63,64,65]. Intriguingly, certain hepcidins also display anticancer properties by suppressing tumor cell proliferation and metastasis, alongside immunomodulatory effects such as cytokine regulation [66]. Additionally, they modulate iron release from hepatocytes and macrophages, further underscoring their dual functionality in bridging innate immunity and systemic iron balance [35,57]. In European sea bass (*D. labrax* L.), hepcidin can prevent nervous necrosis virus-induced mortality by modulating the host immune response, specifically attenuating the virus-triggered pro-inflammatory reaction in the brain while simultaneously promoting antimicrobial peptide expression and leukocyte recruitment [67].

Piscidins are a family of HDPs commonly found in fish, particularly in the gills, muscles, head kidneys, skin, and intestines of bony fish [46]. This family includes various subtypes such as piscidin, pleurocidin, moronecidin, epinecidin, and gaduscidin [46]. These peptides typically feature mature domains of 18–27 amino acids adopting α-helical conformations, though sequence homology among subtypes remains relatively low [68]. Among the well-characterized members, piscidin-1, piscidin-2, and piscidin-3 exhibit distinct structural and functional traits [69]. While piscidin-2 and piscidin-3 share similar lengths in their mature regions, their proprotein convertase cleavage motifs differ markedly: piscidin-2 contains an H-R/K motif at the C-terminus, whereas piscidin-3 harbors an extended R-R-R-H motif [70,71]. Further divergence is observed in their amino acid spacing patterns. In piscidin-2, two conserved glycine residues are separated by 10 amino acids, followed by a 3-residue gap between the second glycine and the cleavage motif. In contrast, piscidin-3 displays a shorter 4-residue interval between glycines and a longer 7–8-residue gap preceding the cleavage site [72,73]. Piscidin-1, distinct from its counterparts, possesses a longer mature peptide with a C-terminal region enriched in hydrophilic residues (aspartic and glutamic acids), potentially enhancing its solubility and interaction with microbial membranes [72,74,75,76]. This structural diversity underscores the functional adaptability of piscidins in combating pathogens across varied aquatic environments.

Defensins are cationic peptides characterized by multiple β-sheet structures stabilized by intramolecular disulfide bonds, playing key roles in innate immunity across species [33]. These peptides are classified into three subgroups (including α-defensins, β-defensins, and θ-defensins) based on variations in disulfide bond connectivity [77]. Notably, θ-defensins are restricted to non-human primates (e.g., rhesus macaques and baboons) and exhibit distinct structural features compared to α- and β-defensins [78]. In fish, only β-defensins have been identified to date, with no reports of α-defensins [79]. While β-defensins are conserved in both humans and fish, their genomic organization differs that human β-defensin genes contain one intron and two exons, whereas fish orthologs possess two introns and three exons [80]. Phylogenetic analyses further categorize fish β-defensins into four subtypes (β-defensin 1–4), each sharing a conserved framework of six cysteine residues [80]. However, subtle structural distinctions exist among subtypes. In β-defensin 1, 3, and 4, the first two cysteines (C1 and C2) are separated by six amino acids, while β-defensin 2 retains only five residues in this interval. Additionally, β-defensin 2 uniquely harbors a P-R-R-Y/L-R motif between its C4 and C5 residues, suggesting functional diversification within this peptide family [81].

Cathelicidins are a family of HDPs characterized by their diverse mature peptide sequences and a conserved N-terminal cathelin domain in their precursor peptides. Unlike many other HDPs, cathelicidins exhibit low sequence homology among their mature peptides, but they share the conserved cathelin domain, which is cleaved by proteases to release the active antimicrobial peptide [47,82]. In mammals, cathelicidins show substantial sequence variability even within a single species. Nevertheless, they share common features as cationic antimicrobial peptides with broad-spectrum activity, and their C-terminal regions are often glycine-rich and strongly positively charged [33]. In fish, the first cathelicidins were identified in Atlantic hagfish (*Myxine glutinosa*), showing structural parallels to mammalian cathelins [83]. Fish cathelicidins are grouped into two types based on the presence or absence of disulfide bonds, with some subtypes displaying high sequence conservation (up to 90%) in specific regions. The third type, discovered in Atlantic cod (*Gadus morhua*), exhibits unique characteristics distinct from other types [84]. The antimicrobial activity of cathelicidins varies significantly between species. For example, cod cathelicidins strongly target Gram-negative bacteria and fungi but show weak activity against Gram-positive strains, while hagfish cathelicidins broadly attack both bacterial types [83]. Similarly, rainbow trout cathelicidins effectively combat *Yersinia ruckeri*, whereas Atlantic salmon versions fail to do so [85]. These differences likely reflect evolutionary adaptations to distinct pathogens. Cathelicidins are tightly regulated by immune signals. In fish embryos, they are expressed early in development and induced by bacterial exposure, mimicking mammalian defense mechanisms [86]. However, lipopolysaccharide (LPS) triggers cathelicidin expression in some species (e.g., coho salmon) but not others, and immune mediators like interleukin (IL)-6 or flagellin can activate their production in specific tissues [87]. In vivo studies confirm their role in infection responses, with time-dependent expression in organs like gills and liver after bacterial challenge [88]. While fish cathelicidin research lags behind mammals, recent findings hint at conserved functions, such as stimulating immune cells to release cytokines like IL-8 [89], suggesting ancient, shared pathways in innate immunity.

NK-lysins are positively charged HDPs produced by immune cells like cytotoxic T lymphocytes and natural killer cells, playing dual roles in pathogen defense and immune regulation [90,91]. In fish, these peptides exist in multiple copies across species. For instance, zebrafish carry four NK-lysin genes, while channel catfish have three [49]. Structurally, NK-lysins belong to the saposin-like protein family, featuring a conserved Saposin-B domain and six cysteine residues critical for their antimicrobial function. Their unique architecture includes five amphipathic α-helical segments folded into a compact, globular shape, enabling interactions with negatively charged microbial membranes [50,92]. Upon binding, NK-lysins disrupt membrane integrity and penetrate cells to damage DNA, effectively neutralizing pathogens. Beyond their broad antibacterial activity, these peptides also exhibit immune-modulating properties and show potential in targeting tumor cells [91,92,93]. This multifunctionality highlights their evolutionary significance as versatile tools in both innate and adaptive immunity.

### 2.2. Mechanisms of Action

**Figure 1:**
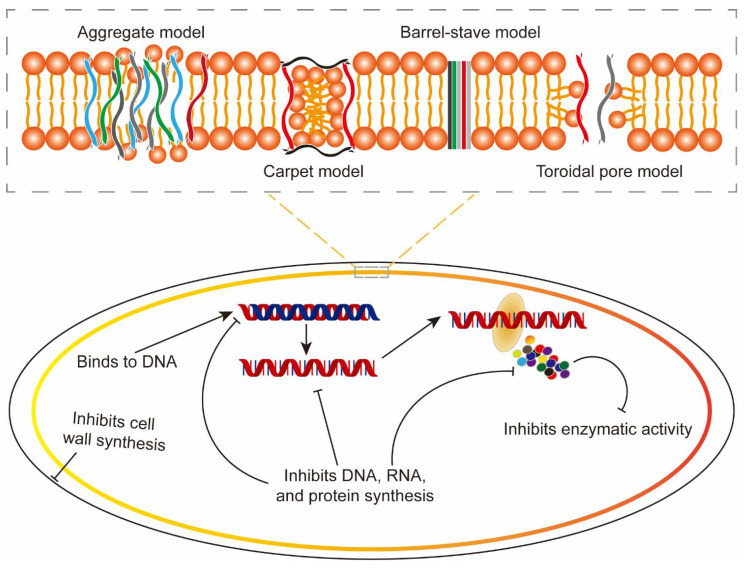
The action mechanism of host defense peptides. The barrel-stave model proposes that HDPs bind to the cell membrane, whereby their hydrophobic domains embed into the lipid bilayer to form a pore. This structure enables the efflux of cellular contents, leading to bacterial death. In contrast, the carpet model involves the accumulation of HDPs on the membrane surface, which increases surface tension and causes membrane deformation and eventual disintegration. The toroidal-pore model describes a process wherein HDPs aggregate and insert into the membrane, inducing curvature in the phospholipid monolayers and resulting in the formation of a toroidal pore approximately 1–2 nm in diameter, ultimately killing the bacterium. Lastly, the aggregated channel model postulates that HDPs bind to phospholipids on the membrane surface, forming peptide-lipid complexes that assemble into channels. These channels facilitate the entry of HDPs into the cell, leading to bacterial death.

HDPs employ multifaceted mechanisms to combat pathogens, combining direct structural disruption with intricate metabolic and immune modulations (Figure 1). At their core, HDPs utilize membrane-targeted actions, where cationic and amphipathic properties drive electrostatic interactions with microbial membranes [94]. This leads to pore formation through models like the barrel-stave mechanism, where α-helical peptides insert vertically to create stable channels (e.g., daptomycin disrupting Gram-positive bacteria), or the toroidal pore model, causing transient membrane curvature and ion leakage (e.g., two fish-derived cathelicidins, salmonoid cathelicidin (CATH_BRALE) and cod cathelicidin (codCath1), depolarizing bacterial membranes) [95]. Shorter peptides may adopt a carpet model, accumulating on membrane surfaces until lipid bilayer collapse occurs, particularly effective against Gram-negative bacteria [96]. In the carpet model, cationic peptides align parallel to the membrane; once a threshold surface density is reached, they disrupt lipid packing and induce positive curvature and transient toroidal pores, ultimately leading to bilayer disintegration. The toroidal-pore model describes a process wherein HDPs aggregate and insert into the membrane, inducing curvature in the phospholipid monolayers and resulting in the formation of a toroidal pore approximately 1–2 nm in diameter, ultimately killing the bacterium [80,97]. Lastly, the aggregated channel model postulates that HDPs bind to phospholipids on the membrane surface, forming peptide-lipid complexes that assemble into channels [98]. These channels facilitate the entry of HDPs into the cell, leading to bacterial death. Some HDPs also induce bacterial agglutination by binding cell wall components like lipopolysaccharides (LPS), promoting phagocytosis and clearance (e.g., NK-lysin, dicentracin and Larimicin_78-102_ enhancing immune responses) [51,99]. Beyond membrane targeting, HDPs interfere with cell wall biosynthesis by inhibiting lipid II-dependent peptidoglycan assembly (e.g., teixobactin and plecatasin) or triggering autolytic enzyme release (e.g., bacitracin) [100]. Once inside cells, they disrupt intracellular processes: binding nucleic acids to block replication and transcription (e.g., peptide 7 (P7) halting *E. coli* DNA synthesis) [101], inhibiting ribosomal activity to suppress protein translation, or targeting energy metabolism by impairing mitochondrial function [102]. Larimicin_78-102_ showed excellent antibacterial and anti-biofilm properties against the three aquatic pathogens (*Vibrio fluvialis*, *Pseudomonas fluorescens*, and *Pseudomonas putida*) by compromising both their outer and inner membranes. This disruption induced adenosine triphosphate (ATP) leakage and elevated intracellular reactive oxygen species (ROS) levels, culminating in bacterial cell death [51]. In several instances, studies in mammals were used to illustrate conserved mechanisms of HDP activity, including membrane disruption, immunomodulatory signaling, and interactions with microbial metabolites. These examples, however, are provided solely as comparative context, and their applicability to teleost fish remains to be validated. While highlighting such parallels is useful for generating hypotheses, direct experimental confirmation in fish is still necessary to establish species-specific mechanisms.

Equally vital is their immunomodulatory role. HDPs recruit immune cells via chemotactic signals and neutralizing endotoxins like LPS to dampen excessive inflammation [103]. Additionally, they enhance vaccine efficacy by priming adaptive immunity, as seen in swine fever vaccines where HDPs boost protective responses [104,105]. Two European sea-bass-derived HDPs, NK-lysin and dicentracin, improved humoral immunity and brain immune-related genes expression in nervous necrosis virus-infected European sea bass [99]. This multitargeted versatility positions HDPs as critical players in host defense, combining direct cytotoxicity with immune system orchestration to counter diverse microbial threats.

## 3. Intestinal Microbiota

In healthy fish, the intestinal microbiota typically exhibits a relatively stable composition dominated by a few major bacterial phyla. Across teleost species, Proteobacteria and Firmicutes are often the most abundant, followed by Fusobacteria, Bacteroidetes, and Actinobacteria [27,106,107]. Although the exact proportions vary by host species, diet, and environment, many studies have reported average relative abundances of approximately 30–50% Proteobacteria, 10–40% Firmicutes, 10–30% Fusobacteria, 5–25% Bacteroidetes, and 1–10% Actinobacteria in healthy individuals [12,18,106,108]. It has been reported that Proteobacteria (from 30 to 50%) and Firmicutes (from 10 to 30%) were the two dominant bacterial phyla in the intestines of the grouper (*Epinephelus coioides*) with autochthonous probiotics intervention [109]. Liu et al. [110] and Shao et al. [111] found that the intestinal microbiota of large yellow croaker (*Larimichthys crocea*) mainly includes Firmicutes (from 30 to 45%), Proteobacteria (from 25 to 45%), and Bacteroidetes (from 10 to 25%). In addition, a high abundance of Proteobacteria (63–74%) followed by Firmicutes (24–35%) were recorded in the intestines of the European sea bass (*D. labrax*) [112]. This core bacterial community contributes to nutrient metabolism, mucosal protection, and immune regulation, serving as a reference framework for identifying dysbiosis-related alterations.

The intestinal microbiota barrier plays a critical role in maintaining a stable microenvironment in the intestine, preventing harmful microbes and toxins from entering the body, and protecting the intestinal mucosa from damage [113,114,115]. Unlike terrestrial vertebrates, the intestinal microbiota of fish is predominantly composed of aerobic, facultative anaerobic, and obligate anaerobic bacteria. Research indicates that each gram of fish intestinal contents contains approximately 10^7^ to 10^11^ bacteria [116], with Proteobacteria, Bacteroidetes, Firmicutes, Fusobacteria, and Actinobacteria being the core phyla [18,106,108,115,117]. Probiotics can occupy ecological niches in the intestine, thereby inhibiting the growth of harmful microbes. They also contribute to the breakdown of food into absorbable small molecules such as amino acids, fatty acids, polyphenols, and minerals [106,109,117]. These molecules promote the growth and repair of intestinal epithelial cells, enhancing overall growth and metabolism. Additionally, probiotics strengthen the intestinal physical barrier function [116,118]. For example, *B. siamensis* positively with ZO-1, occludin, claudin-b, and E-cadherin expressions in spotted seabass (*L. maculatus*) [27], while *B. amyloliquefaciens* improved intestinal morphology and microbial community [119]. The intestinal microbiota also modulates immune responses by signaling through immune cells to promote immune tissue maturation, antibody production, T cell differentiation, and macrophage activation [116,120,121]. Disruption of the intestinal microbiota barrier can lead to dysbiosis, mucosal damage, and immune dysfunction, potentially causing inflammation, autoimmune diseases, and metabolic disorders [107,122]. Therefore, maintaining the stability and integrity of the intestinal microbiota barrier is essential for fish health.

The intestine exhibits a complex and dynamic interaction between the host and the microbiota. On one hand, the intestinal mucosa serves as an effective barrier against pathogenic invasion through both cellular and humoral immune mechanisms. A variety of immune effectors collectively shape the composition and spatial distribution of the intestinal microbiota in fish [106,109]. On the other hand, commensal bacteria are essential for the development and maintenance of immune homeostasis in fish [8]. This balanced coexistence is underpinned by a multi-layered intestinal barrier system in fish, which includes the epithelial cells, the mucus layer, the mucosal immune system, and the resident microbiota [6]. The intestinal epithelial barrier constitutes the first line of defense against microorganisms and harmful agents in fish, employing a range of protective mechanisms that form a sophisticated innate immune network. Epithelial cells express pattern recognition receptors (PRRs) that monitor the intestinal microbiota by recognizing pathogen-associated molecular patterns (PAMPs) or microbe-associated molecular patterns (MAMPs) [123]. These molecular motifs are highly conserved across microorganisms. Research indicates that intestinal microbiota changes were significantly related with stimulating the three PRR (RIG-like receptor, NOD-like receptor, and Toll-like receptor) signaling pathways in *E. fuscoguttatus* × *E. lanceolatus* [124]. Ligand binding to PRRs triggers rapid and sustained defense responses, including the production and secretion of HDPs, signaling molecules, and mucins [18].

Key PRRs involved include transmembrane Toll-like receptors (TLRs) and intracellular nucleotide-binding oligomerization domain (NOD)-like receptors. PRR activation engages signaling cascades such as those mediated by myeloid differentiation primary response 88 (MyD88), mitogen-activated protein kinases (MAPKs), and nuclear factor kappa B (NF-κB) in fish species, such as spotted seabass (*L*. *maculatus*) [125] and grouper (*E. coioides*) [126]. These pathways orchestrate efforts to eliminate threats and facilitate the recruitment of inflammatory cells through chemokine signaling, further amplifying NF-κB-mediated responses. Recent advances in understanding PRR functions and innate recognition mechanisms have shifted research focus from acute anti-pathogen immunity to the maintenance of commensal homeostasis.

Through PRR-mediated surveillance, intestinal epithelial cells rapidly produce HDPs, defending against pathogens and preventing dysbiosis and bacterial translocation. In turn, a balanced microbiota and an intact mucus layer modulate PRR activity, maintaining pro-inflammatory cytokines at low baseline levels and thereby promoting equilibrium between microbes and barrier function. Disruption of any component, such as reduced expression of HDPs, can lead to dysbiosis, impaired barrier function, and subsequent disease in fish.

## 4. Interaction Between HDPs and Intestinal Microbiota

### 4.1. HDPs-Mediated Intestinal Microbiota

HDPs act as molecular architects of intestinal microbial communities in teleosts, with species-specific adaptations reflecting evolutionary pressures. In common carp (*Cyprinus carpio*), hepcidin-2 expression increases post-infection with *Aeromonas hydrophila*, selectively reducing iron-dependent pathogens while preserving *Cetobacterium somerae* populations [127]. This bacterium, critical for thiamine biosynthesis, colonizes the carp intestine in densities exceeding 10^9^ CFU/g, highlighting HDPs’ role in maintaining mutualistic relationships. Transcriptomic studies reveal that hepcidin upregulates microbial genes involved in iron sequestration and amino acid biosynthesis, fostering metabolic synergy [128,129,130].

Spatially, HDPs guide microbial biofilm formation [131]. Piscidin-3 disrupts *Flavobacterium columnare* biofilm matrices by binding to LPS, reducing adhesion by 70% [75]. Meanwhile, *B. subtilis* supplementation enhances HDP-mediated protection. For instance, in challenged catfish, *Bacillus* exopolysaccharides complex with piscidin-3, extending its half-life and improving clearance of *Edwardsiella ictalurid* [132]. Microbiota profiling shows that catfish HDPs also shape phage-bacteria interactions, favoring temperate phages that lyse *Aeromonas* spp. while sparing probiotics [133,134]. This multitrophic regulation ensures microbial stability even in high-stress aquaculture environments.

### 4.2. Feedback Regulation of HDPs Expression by Intestinal Microbiota

**Figure 2:**
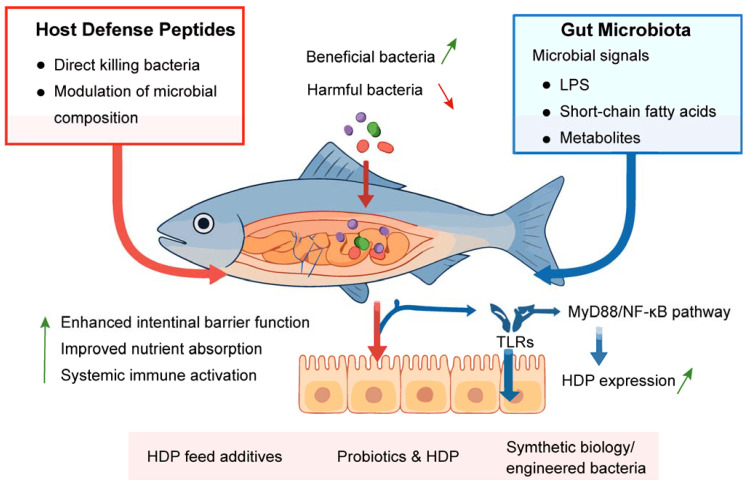
Schematic diagram of crosstalk between intestinal microbiota and host defense peptides in fish. LPS, lipopolysaccharide; TLRs, toll-like receptors; HDPs, host defense peptides.

The intestinal microbiota exerts precise control over HDPs in teleosts through metabolite signaling and immune priming (Figure 2). In Atlantic salmon (*S. salar*), *Ruminococcaceae*-derived butyrate activates G-protein-coupled receptor 43 (GPR43) on enterocytes [135], inducing histone acetylation at the *defb1* promoter and increasing β-defensin production [136]. Conversely, in spotted seabass (*L. maculatus*), *B. siamensis* secretes lipoteichoic acid (LTA) or peptidoglycan (PGN) fragments that engage toll-like receptors, triggering p38 MAPK/NF-κB pathways and HDPs (including β-defensin, hepcidin-1, NK-lysin and piscidin-5) induction [18,125]. Further analysis reveals that increased HDPs reciprocally upregulate anti-inflammatory cytokines, suppress pro-inflammatory cytokines and apoptosis, and reshape intestinal microbiota, protecting spotted seabass from soybean meal-induced enteritis [18].

Pathogen-driven feedback loops further fine-tune HDP responses. In rainbow trout (*Oncorhynchus mykiss*), *A. salmonicida* LPS induces a biphasic defensin response: *defb2* peaks at 4 h to combat acute infection, while *cathelicidin-B* rises at 24 h to resolve inflammation via alternatively activated macrophage (M2) macrophage polarization [137,138]. This dysregulation highlights the microbiota’s role as both guardian and vulnerability in aquaculture.

### 4.3. Dynamic Homeostasis in Microbiota and HDP Crosstalk

The microbiota–HDP axis in fish demonstrates remarkable plasticity in adapting to environmental challenges. During viral hemorrhagic septicemia virus (VHSV) outbreaks in Atlantic salmon (*S. salar*), *Piscirickettsia salmonis* infection reduces *Cetobacterium* abundance, lowering short-chain fatty acids (SCFA) production and hepcidin expression [139]. This creates a pro-inflammatory state characterized by neutrophil infiltration and *Vibrio* overgrowth. However, recovery is possible. Probiotic *B. amyloliquefaciens* supplementation restores *Cetobacterium* and SCFAs, and improving survival [119]. Mechanistically, *Bacillus* secretes surfactin, which binds *Piscirickettsia* LPS, blocking TLR4 activation and preventing excessive HDP secretion [140].

Temperature shifts also challenge this equilibrium. In Nile tilapia (*Oreochromis niloticus*) acclimated to 34 °C, intestinal anaerobes overproduce LPS, triggering TLR4 hyperactivation and excessive HDP secretion [141]. This depletes *Lactobacillus* populations, creating a pro-inflammatory state with elevated interleukin (IL)-1β and mucosal ulceration [142,143]. However, the microbiota evolves countermeasures. *B. licheniformis* in heat-stressed tilapia increases exopolysaccharide production, physically shielding bacteria from HDPs while secreting epidermal growth factor-like proteins to repair mucosal damage [144]. Metagenomic studies reveal that heat-adapted *Bacillus* strains carry mutations in LPS biosynthesis genes (e.g., *waaL*), reducing negative charge and HDP binding [145]. This arms race between microbes and peptides underscores their coevolutionary role in maintaining health in dynamic aquatic ecosystems, with implications for climate-resilient aquaculture. Future research should explore clustered regularly interspaced short palindromic repeats (CRISPR)-based HDP optimization to enhance stress resilience while preserving microbial diversity.

### 4.4. Non-Bacterial Intestinal Communities and Knowledge Gaps

Although the present review focuses primarily on bacterial taxa, it is important to acknowledge that the fish intestinal harbors other microbial components, including fungi (the mycobiome), archaea, and protozoa. Emerging studies using amplicon sequencing have detected diverse fungal lineages in the intestines of freshwater and marine teleosts, with Candida, Debaryomyces, and Pichia reported as recurrent genera in fish [146,147]. However, direct experimental evidence linking HDPs to the modulation of the mycobiome in fish is currently lacking. This represents a significant knowledge gap, as cross-kingdom interactions between fungi, bacteria, and host immunity could influence mucosal homeostasis, pathogen resistance, and nutritional outcomes. Future research should therefore prioritize targeted mycobiome-focused studies—combining amplicon/metagenomic profiling with functional assays—to clarify whether and how HDPs shape fungal communities, and to explore potential synergies or trade-offs with bacterial counterparts. Explicitly addressing these non-bacterial components will help to build a more holistic understanding of intestinal ecosystem regulation in fish.

## 5. Applications of the Crosstalk Mechanism in Fish

The intricate crosstalk between intestinal microbiota and host defense peptides (HDPs) presents promising opportunities for advancing sustainable aquaculture by enhancing disease resistance, improving stress tolerance, and optimizing nutritional strategies. Below, we elaborate on the practical applications of this mutualistic interaction, supported by recent scientific evidence and technological innovations.

### 5.1. Disease Prevention and Control Through Microbiota–HDP Synergy

One of the most impactful applications involves modulating the microbiota to enhance HDP-mediated immunity, thereby reducing reliance on antibiotics. Probiotic supplementation in aquafeeds has emerged as a key strategy. For example, dietary administration of *Lactiplantibacillus plantarum* E2 to juvenile yellow croaker (*Larimichthys crocea*) was shown to upregulate the expression of HDPs in the intestinal mucosa [148]. This transcriptional activation coincided with an approximately 62% reduction in mortality following challenge with *Vibrio parahaemolyticus*, compared to the control group. The mechanism involves probiotic-induced activation of the TLR4/NF-κB signaling pathway, which not only stimulates HDP production but also suppresses pro-inflammatory cytokine secretion (e.g., IL-6 and tumor necrosis factor (TNF)-α), creating a balanced immune response that prioritizes pathogen elimination over excessive inflammation [149]. The relative expressions of NK-lysin and Galectin-3 were significantly up-regulated in yellow drum (*Nibea albiflora*) intestines fed with *Enterococcus faecalis* and *B. subtilis*, which are conducive to maintain intestinal microbiota homeostasis and improve red-head disease resistance [108].

In European sea bass (*D. labrax*), co-culture experiments revealed that *B. subtilis* secretes short-chain fatty acids (SCFAs) [150], particularly butyrate, which enhance the epithelial barrier function by increasing tight junction protein expression (e.g., zonula occludens (ZO)-1) alongside HDP induction [151]. This dual effect not only restricts pathogen adhesion (e.g., *A. hydrophila*) but also creates a hostile environment for invading microbes through HDP-mediated membrane permeabilization. Furthermore, it has been demonstrated that probiotic-treated fish exhibit a 30% lower colonization density of pathogenic vibrios in the intestine, translating to a 40% reduction in clinical disease incidence during high-density farming conditions [152].

### 5.2. Mitigating Environmental Stress

Aquaculture species are frequently exposed to abiotic stresses, such as water pollution (e.g., per- and polyfluoroalkyl substances, PFAS), heavy metals (e.g., cadmium), and thermal fluctuations, which disrupt intestinal microbiota homeostasis and impair HDP production. Recent studies highlight the protective role of the microbiota–HDP crosstalk in alleviating such stresses. For instance, when zebrafish larvae were exposed to PFBS (a PFAS analog), intestinal microbiota dysbiosis occurred, characterized by a 50% decrease in beneficial *Bacteroidetes* and a concurrent downregulation of *β-defensin* and *hepcidin* [153]. However, pre-treatment with *Lactobacillus rhamnosus* GG (LGG) restored microbial diversity, increased SCFA production, and upregulated HDP genes, effectively mitigating sepsis-induced intestinal epithelial damage (e.g., reduced villus atrophy and mucus layer thinning) [154].

### 5.3. Precision Regulation Through Targeted Microbiota–HDPs Interventions

Advances in gnotobiotic models (e.g., germ-free zebrafish and axenic shrimp larvae) have enabled researchers to dissect strain-specific interactions between microbiota and HDPs, paving the way for precision aquaculture strategies [155,156]. For example, mono-colonization of germ-free zebrafish with *Plesiomonas shigelloides* was shown to uniquely induce the expression of *hepcidin-2* through iron sequestration mechanisms [157], while *Lactococcus lactis* preferentially upregulated *β-defensin-1* via SCFA-mediated histone deacetylase inhibition [158]. Moreover, it has been reported that autochthonous probiotics could modulate intestinal HDPs expressions and microbial community in spotted seabass (*L. maculatus*) [27], yellow drum (*N. albiflora*) [108], and grouper (*E. coioides*) [126], ultimately enhancing fish immunity. Such mechanistic insights allow for the design of “customized probiotics” that target specific HDP pathways based on the species’ genetic background and environmental challenges.

Another frontier is the development of HDP-mimetic peptides or microbial consortia that mimic natural crosstalk. Synthetic peptides derived from fish *piscidins*, such as piscidin (PIS)-1, have been shown to selectively disrupt pathogenic biofilms (e.g., *Vibrio harveyi*) while promoting the growth of beneficial *Bacteroides* in in vitro co-culture systems [76,159]. When encapsulated in chitosan nanoparticles and delivered orally to tilapia (*O. niloticus*), these mimetic peptides increased intestinal microbiota diversity, enhanced HDP gene expression, and improved feed conversion efficiency, demonstrating their potential as eco-friendly alternatives to antibiotics [160].

Furthermore, metabolomic profiling has identified key microbial metabolites, such as indole-3-propionic acid (IPA) and propionate, that act as signaling molecules between microbiota and HDP-producing cells [161]. In a proof-of-concept study, exogenous supplementation of IPA to Atlantic salmon (*S. salar*) juveniles upregulated *cathelicidin* expression in the intestine, leading to a reduction in *Yersinia ruckeri* infection severity without altering the core microbiota composition [162]. This suggests that targeted metabolite intervention could offer a non-invasive approach to enhance host defense without disrupting microbial balance.

### 5.4. Effects on Growth Performance

Beyond their roles in disease resistance, HDPs and the intestinal microbiota also exert measurable impacts on fish production traits. Several studies have reported that modulation of HDPs, either through dietary supplementation, genetic overexpression, or probiotic-mediated stimulation, can improve growth performance by enhancing nutrient absorption and reducing pathogen-induced energy losses [108,109]. Likewise, beneficial microbiota promoted by HDP activity, such as *Lactobacillus*, *Bacillus*, and *Cetobacterium*, have been associated with improved feed conversion ratios, vitamin synthesis, and short-chain fatty acid production, all of which support efficient metabolism and weight gain [117,144]. In contrast, dysbiosis characterized by opportunistic *Desulfovibrio* overgrowth often coincides with reduced growth rate, poor feed utilization, and heightened disease susceptibility [107]. These observations underscore that HDP–microbiota crosstalk is not only an immunological safeguard but also a key determinant of aquaculture performance. A more integrative understanding of these interactions could enable the design of dietary, genetic, and microbial strategies that simultaneously strengthen host immunity and optimize production efficiency in farmed fish.

## 6. Conclusions

HDPs play a dual role in fish immunity by directly inhibiting pathogens and shaping the intestinal microbiota. Evidence shows that HDPs favor beneficial taxa such as *Lactobacillus*, *Bacillus*, *Cetobacterium*, and *Lactococcus*, while microbial signals in turn regulate HDP expression, establishing a reciprocal communication that contributes to intestinal homeostasis and disease resistance. However, important gaps remain. The mechanisms by which HDPs modulate community composition are incompletely understood, and little is known about their influence on non-bacterial components such as fungi and archaea. Translational applications also face challenges, including scalable production, stability during feed processing, and ecological safety.

Future work should prioritize standardized multi-omics studies in aquaculture species, development of engineered microbial systems for in situ HDP delivery, and combinatorial strategies integrating HDPs with probiotics, prebiotics, or phytogenics. Expanding research to cross-kingdom interactions will further clarify the ecological scope of HDP activity. Addressing these potential problems will advance both mechanistic understanding and practical solutions, helping to build resilient microbiota, strengthen fish health, and reduce antibiotic dependence in fish aquaculture.

## Figures and Tables

**Table 1:** Major host defense peptide (HDP) families identified in teleost fish, their structural characteristics, representative examples, expression sites, and principal functions.

HDP Family	Typical Length/Structural Features	Representative Species	Primary Tissue Expression	Principal Functions
Hepcidins	20–25 aa mature peptide; 8 conserved cysteines forming β-sheet	Zebrafish (*Danio rerio*), tilapia (*Oreochromis niloticus*), rainbow trout (*Oncorhynchus mykiss*)	Liver, intestine, gill, kidney	Iron homeostasis; broad-spectrum antimicrobial activity; immunomodulation
β-defensins	30–45 aa; six conserved cysteines forming three disulfide bonds	Zebrafish, channel catfish (*Ictalurus punctatus*), grass carp (*Ctenopharyngodon idella*)	Intestine, gill, skin, spleen	Membrane disruption; chemotactic activity; regulation of adaptive immunity
Piscidins	20–40 aa; amphipathic α-helical structure	Hybrid striped bass (*Morone saxatilis × M. chrysops*), European sea bass (*Dicentrarchus labrax*)	Mast cells, gill, intestine, skin	Potent antimicrobial activity (Gram+/Gram−, fungi, parasites); wound healing
Cathelicidins	25–37 aa mature peptide; highly variable α-helical/β-sheet domains	Atlantic salmon (*Salmo salar*), rainbow trout	Head kidney, spleen, mucosal tissues	Direct antimicrobial activity; immune cell recruitment; modulation of inflammation
NK-lysins	74–78 aa; saposin-like domain structure	Channel catfish, turbot (*Scophthalmus maximus*)	Head kidney, spleen, intestine, gill	Cytotoxicity against tumor and pathogen-infected cells; bacterial killing

## Data Availability

This review paper does not use any data. The required files were all obtained from the internet.
